# Effects of Sheltering Conditions on Serum Biochemical and Stress Hormone Profiles of Lamb During Cold Exposure

**DOI:** 10.3390/ani16081146

**Published:** 2026-04-09

**Authors:** Xintong Li, Zhipeng Han, Xiao Jin, Bo Wang, Dengsheng Sun, Wenliang Guo

**Affiliations:** 1 College of Animal Science, Inner Mongolia Agricultural University, Zhaowuda Road 306, Hohhot 010018, China; lixintongaa@163.com (X.L.); hzp4639@163.com (Z.H.); 18686197338@163.com (W.G.); 2 State Key Laboratory of Animal Nutrition and Feeding, College of Animal Science and Technology, China Agricultural University, Beijing 100193, China; wangbo123@cau.edu.cn; 3 Animal-Centered Environments Group, Department of Biosystems and Engineering, Swedish University of Agricultural Sciences, P.O. Box 190, 23422 Lomma, Sweden; dengsheng.sun@slu.se

**Keywords:** lamb, sheltering conditions, cold exposure, blood indices

## Abstract

Long-term cold exposure reduces livestock welfare and productivity in Inner Mongolia. This study assessed cold stress effects on 60 two-month-old female Dorper × Mongolia lambs allocated to four sheltering conditions (*n* = 15): indoor pens with enclosed housing (IP), outdoor pens (OP), house with playground pens (OPP), and polytunnel pens (PP). Compared with IP, OP exhibited significantly lower temperature, humidity, CO_2_ concentration, NH_3_ concentration, and WCI, and significantly higher wind speed and solar radiant heat (*p* < 0.001). Humidity, CO_2_ concentration, and NH_3_ concentration in PP was lower than in IP, but higher than in OP (*p* < 0.001); temperature, wind speed, and WCI did not differ significantly between PP and IP. ADG was significantly lower in OP and OPP than in IP (*p* < 0.001), whereas PP did not differ from IP. F:G was higher in OP than in IP and PP (*p* = 0.040). Feeding duration had significant effects on ACTH, leptin, T_3_, T_4_, TP, urea, TG, NEFA, LDL, and HDL concentrations. Rearing environment significantly affected GLU, ALB, LDH, and TG. Feeding duration × sheltering conditions interaction significantly influenced ACTH, TP, ALB, urea, LDH, TG, LDL, and HDL. OP induced cold stress and dysfunction, while IP and PP produced milder responses. PP raised indoor temperatures substantially, and is thus optimal for winter lamb production.

## Simple Summary

Cold stress remains a key constraint in lamb production in cold regions. In this study, the serumal biochemical and stress hormone profiles were compared in lambs housed in an enclosed house with a playground, outdoor pens, indoor pens, and a polytunnel. Compared with lambs in other enclosures, the adverse effects of cold stress on lambs in the polytunnel pens were reduced. We conclude that the house with the polytunnel is best for lamb welfare.

## 1. Introduction

Low temperature exposure redirects nutrients away from productive functions toward cold resistance, reducing nutritional efficiency while increasing lipid utilization to maintain energy metabolism, ultimately compromising both profitability and welfare of livestock in cold regions [1,2,3]. Sheep have dense fleece, the interstices of which trap large volumes of still air, creating a natural insulating layer that reduces conductive and radiative heat loss [4,5]. However, wind speed and humidity strongly affect convective and evaporative heat exchange in animals [6]. Mejdell et al. highlighted that the combination of cold rain and strong wind presents a major thermoregulatory challenge for horses in winter [7]. In wet, cold conditions, water displaces the air within wool fibers; because water’s thermal conductivity is 25 times that of still air [8], conductive heat loss increases sharply and sheep become much more susceptible to cold stress. Piirsalu et al. [9] examined sheep in Estonia during winter, when temperatures reached as low as −23 °C. While most animals remained outdoors, the proportion seeking shelter declined significantly as ambient temperature fell, relative humidity increased, and the wind-chill index (WCI) rose (*p* < 0.001). These findings suggest that improving housing to reduce WCI and modestly increase ambient temperature would promote shelter use and likely enhance welfare. Low temperature and strong wind speed decreased average daily gain in lambs [1]. When lambs experience cold stress, core temperature is preserved through physiological adjustments [3]. Short-term cold exposure activates the sympathetic nervous system (SNS), increases catecholamine release, and raises metabolic rate and thermogenic capacity [10]. The prolonged exposure elicits cold stress to activate the hypothalamic–pituitary–thyroid (HPT) and hypothalamic–pituitary–adrenal (HPA) axes, causing secretion of cortisol, triiodothyronine (T_3_), and thyroxine (T_4_), which elevates the metabolic rate to enhance thermogenesis [11]. Therefore, non-esterified fatty acids (NEFA) and lactate dehydrogenase (LDH) in blood increase to promote lipolysis and energy production and to support sustained heat generation [12]. In addition, cold environments enhance fermentation of short-chain fatty acids (SCFAs) in the ovine cecum, supplying additional substrates for thermogenesis [13]. However, chronic cold stress impairs growth performance and suppresses immune function in lambs, negatively affecting winter livestock production [14,15].

Inner Mongolia, located in northern China, is a major lamb-producing region. Stunted growth in lambs, diseases, and mortality are caused by a long and frigid winter, which generally lasts from October to the next March, with temperatures remaining around −20 °C for an extended period and even dropping below −40 °C during the coldest spells [15,16]. The environmental temperature falls below the thermoneutral zone, causing chronic cold stress in lambs [17]. Lamb growth stunting, disease, and mortality caused by chronic cold stress constitute the primary breeding losses in Inner Mongolia during the winter months [17]. A study in Australia found that using shelters reduced twin lamb mortality by 17.5% and lowered the incidence of dystocia and other ewe diseases [18]. Providing sheltered enclosures to protect lambs from cold exposure in winter is a particularly important measure to ensure lamb production and animal welfare [15,19]. Kalyan et al. described several standardized lamb housing types: open sheds with roofs and fences; semi-open sheds with one wall reaching animal height and the rest open to the roof; and enclosed sheds with roofs, walls, and louvered vents [20]. Those designs were developed for tropical climates. In this study, we adapted several of these shed types for the Inner Mongolia region and, taking local conditions into account, added a greenhouse-style shelter. We speculate that the use of IP sheds can increase the temperature inside the sheds by concentrating short-wave solar radiation, thereby reducing the cold stress response of lambs. Our aim was to identify housing configurations suitable for temperate to cold climates and to improve lamb welfare in Inner Mongolia.

Therefore, this study selected four commonly used winter sheltering conditions in Inner Mongolia and compared the blood biochemical indicators and stress hormones in lambs among the four sheltering models to determine appropriate winter sheltering for the region.

## 2. Materials and Methods

### 2.1. Animal and Experiment Sheltering Conditions

Referring to the experimental designs of Yanar et al. [21], Fereig et al. [22], Laburn et al. [23], and Wang et al. [16], this experiment selected 60 two-month-old healthy Dorper × Mongolia female lambs, with average body weight of 16.42 ± 2.52 kg and fleece length of 50 mm, which were randomly selected from a flock of 150 on the Hailiutu experimental farm in Inner Mongolia Agricultural University (Hohhot, Inner Mongolia, China). The experiment ran from December through January, the coldest period in the region. During this interval, the mean temperature was −9.13 ± 4.92 °C and the mean relative humidity was 50.42 ± 15.3%. The lambs were randomly assigned to four sheltering condition groups, each had five pens with three lambs (*n* = 15 per group, N = 60 in total).

The sheltering conditions groups were as follows: indoor pens with enclosed housing (IP), outdoor pens (OP), house with playground pens (OPP), and polytunnel pens (PP). The dimensions of each individual pen were 4 × 5 m^2^. The specific design of the pens was as follows: IP group, which had a closed lamb house with four breeze block walls, a door and window on the front wall, and an opaque roof; OP group, where lambs were kept in pens in a relatively exposed open area with each pen delineated by a metal mesh fence without any shelter; and OPP group, which had a closed lamb house with windows and a playground. The lambs were free to choose whether they were inside or outside the house. The PP group were in individual pens in a metal-framed construction with translucent plastic sides and roof. None of the pens have any artificial lighting or ventilation equipment.

### 2.2. Feeding and Diets

During the experiment, the lambs were fed twice a day (at 08:00 and 16:00), and had free access to water. The composition and nutrient levels of the experimental diet are shown in Table 1.

### 2.3. Measurements

#### 2.3.1. Environmental Parameters

Environmental temperature and humidity were recorded with a ZJI-2A Self-Recording Temperature and Humidity Recorder (Shanghai Longtuo Equipment Co., Ltd., Shanghai, China) suspended at 0.5 m in the center of the lamb stable. Wind speed was measured three times daily at 08:00, 14:00, and 20:00 using a Testo 416 Digital Mini Vane Anemometer (Testo, 99 Washington Street, Melrose, MA, USA). The WCI calculation formula is WCI = 13.12 + 0.62*T* − 13.17 × [WS]^0.16^ + 0.4*T*[WS]^0.16^ [24], where WS = wind speed in km/h and *T* = temperature in °C. Ammonia and carbon dioxide concentrations were measured daily at 08:00, 14:00, and 20:00 using gas detector tubes (Beijing Beike Oasis Safety Environmental Technology Co., Ltd., Beijing, China). Thermal radiation was measured concurrently with an MR-5 radiometer. This study was a continuation of our prior research [15].

#### 2.3.2. Feeding Indices

We recorded daily supplied feed and residual feed for each lamb to calculate average daily feed intake (ADFI). We measured body weight of each lamb on days 0, 14, and 28, and used these values to calculate average daily gain (ADG) and feed-to-gain ratio (F:G).

#### 2.3.3. Blood Indices

On days 7, 14, 21, and 28 of the trial, just before the 06:00 morning feeding, blood was collected from each lamb by jugular venipuncture and placed into both plain vacuum tubes and EDTA anticoagulant tubes. After centrifugation at 3000× *g* for 20 min, serum was separated, aliquoted, and stored at −20 °C. Cortisol (JRXW740406, 10 ng/mL–160 ng/mL, CV < 15%), leptin (RX800346SH, 0.5 ng/mL–16 ng/mL, CV < 10 %), T_3_ (JRXW740456, 0.5 nmol/L–8 nmol/L, CV < 15%), T_4_ (JRXW740296, 15 nmol/L–240 nmol/L, CV < 15%), adrenocorticotropic hormone (ACTH) (RX2D749126, 12.5 pg/mL–400 pg/mL CV < 15%), and NEFA (RXWB0836-96, CV < 15%) were measured using commercial sheep-specific ELISA kits (Ruixin Biological Technology Co., Ltd., Quanzhou, China) following the manufacturer’s instructions. Additional serum parameters, including total protein (TP), albumin (ALB), triglycerides (TG), urea, glucose (GLU), high-density lipoprotein (HDL), low-density lipoprotein (LDL), and LDH, were analyzed using a HITACHI 7020 automatic biochemical analyzer (Hitachi High-Tech Corporation, Tokyo, Japan).

### 2.4. Statistical Analysis

Experimental data were collated in Microsoft Excel 2016. Environmental and feeding indices were analyzed via one-way ANOVA using the GLM procedure of SAS 9.2 (SAS Institute, Inc., Cary, NC, USA); Duncan’s multiple range test (DMRT) was used for post hoc comparisons between groups, with statistical significance set at *p* < 0.05.

Stress hormone and blood biochemistry data were analyzed using R 3.6.1 software. Linear mixed models were fitted via the lmer function of the lmerTest package version 3.1-0 [25]. Given the repeated-measures design and interdependence among animals in the same pen, individual animal and pen number were designated as random effects. Two variables (ACTH and ALB) were poorly fitted when individual animals were included as a random effect; thus, weekly means for each individual were calculated, and models were re-run with sheltering conditions as the fixed effect and pen number as the only random effect. Several variables (ACTH, ALB, Leptin, NEFA, T_3_, T_4_, TG, TP) were log-transformed prior to analysis.

Additionally, one-way ANOVA (SAS 9.2) followed by DMRT was performed for post hoc comparisons across different sheltering conditions at a single time point. Similarly, the same approach was adopted for comparisons across different rearing times within a single sheltering condition. All data are presented as mean ± standard deviation (SD). Statistical significance was defined as *p* < 0.05, and extremely significant difference as *p* < 0.01.

## 3. Results

### 3.1. Environmental Parameters

**Figure 1:**
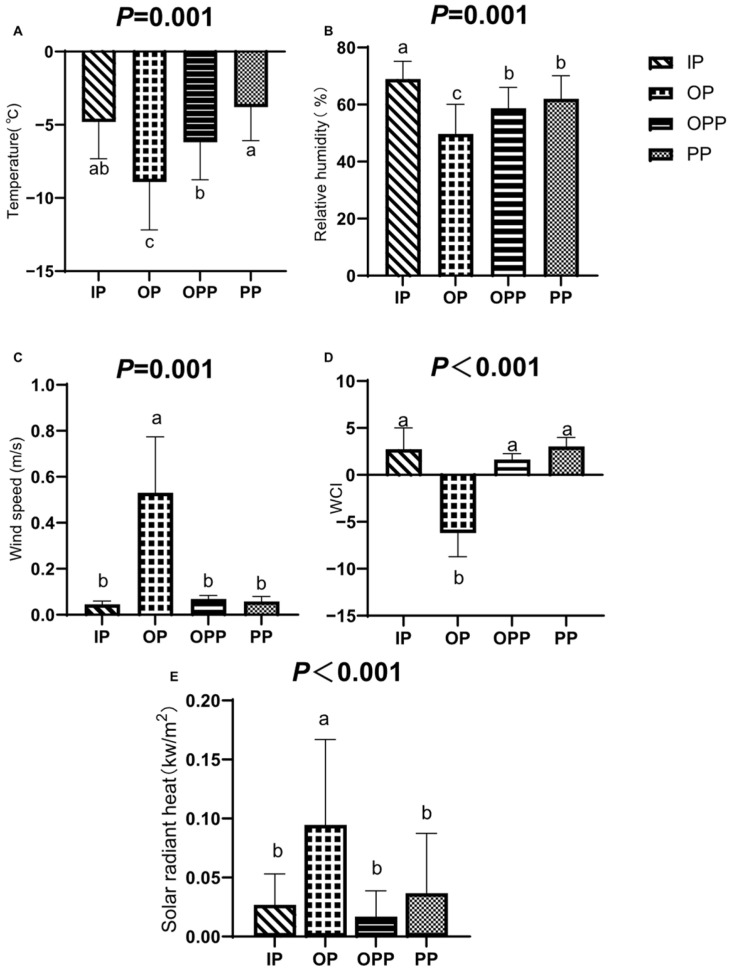
Environmental indicators of sheltering conditions during the test period. Note: (**A**) temperature differences among sheltering conditions; (**B**) relative humidity differences among sheltering conditions; (**C**) wind speed differences among sheltering conditions; (**D**) wind cooling index (WCI) differences among sheltering conditions. The WCI calculation formula is WCI = 13.12 + 0.62*T* − 13.17 × [WS]^0.16^ + 0.4*T*[WS]^0.16^, where WS = wind speed in km/h and *T* = temperature in °C. (**E**) Solar radiant heat differences between different sheltering conditions. a, b: Lowercase letters (a, b, c) indicate significant differences between different sheltering conditions (*p* < 0.05). *p* < 0.05 indicates a significant difference, *p* < 0.01 indicates a highly significant difference, and 0.05 ≤ *p* < 0.10 was considered to show a trend toward a difference. *n* = 15 per group.

Figure 1 shows that ambient temperature was significantly lower in the OP and OPP groups than in the IP and PP groups (*p* = 0.001), with no significant difference between IP and PP. Relative humidity in OP and OPP was significantly lower than in IP, while PP had significantly higher humidity than OP (*p* = 0.001). Wind speed was greater in OP than in the other three groups (*p =* 0.001). The wind chill index (WCI) in OP was significantly lower than in the other groups (*p* < 0.001). Meanwhile, solar radiant heat was significantly greater in the OP group than that in the other three groups (*p* < 0.001). Furthermore, the solar radiant heat of the PP group was the second-highest among the three shelter conditions.

**Figure 2:**
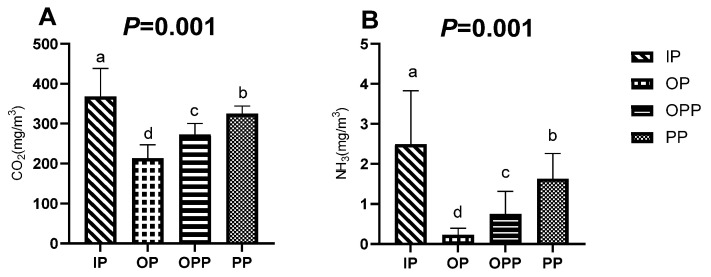
Gas concentration indicators of sheltering conditions during the test period. Note: (**A**) concentration of CO_2_ differences among sheltering conditions; (**B**) concentration of NH_3_ differences among sheltering conditions. a, b: Lowercase letters (a, b, c, d) indicate significant differences among sheltering conditions (*p* < 0.05). *p* < 0.05 indicates a significant difference, *p* < 0.01 indicates a highly significant difference, and 0.05 ≤ *p* < 0.10 was considered to show a trend toward a difference. *n* = 15 per group.

Figure 2 shows that CO_2_ concentration was highest in the IP group, which was significantly greater than that observed in all other groups (*p* = 0.001). The CO_2_ concentrations in the shed decreased in the following order: IP > PP > OPP > OP, with all pairwise differences being highly significant. The NH_3_ concentration was significantly higher in the IP and PP groups than in the OP and OPP groups, with the OPP group showing higher NH_3_ than the OP group (*p* = 0.001).

### 3.2. Growth Performance

**Figure 3:**
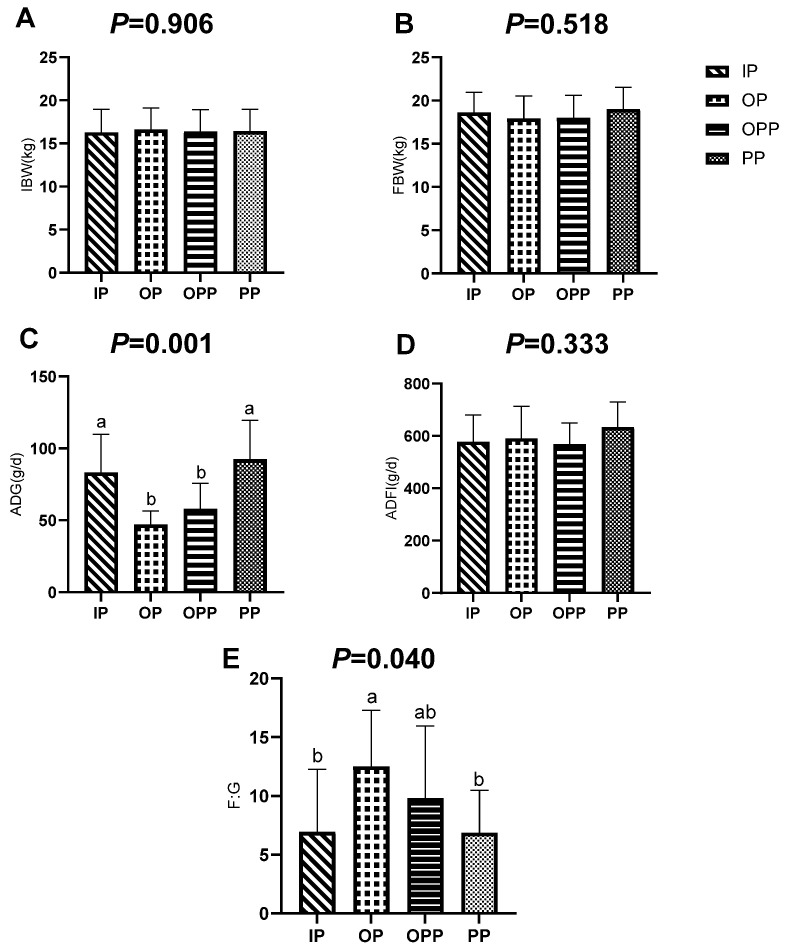
Effects of sheltering conditions on growth performance of female lambs. Note: (**A**) IBW differences among sheltering conditions; (**B**) FBW differences among sheltering conditions; (**C**) ADG differences among sheltering conditions; (**D**) ADFI differences among sheltering conditions; (**E**) F:G differences among sheltering conditions. IBW: initial body weight, FBW: final body weight, ADG: average daily gain, ADFI: average daily feed intake, F:G: feed-to-gain ratio. a, b: Lowercase letters (a, b) indicate significant differences among sheltering conditions. *p* < 0.05 indicates a significant difference, *p* < 0.01 indicates a highly significant difference, and 0.05 ≤ *p* < 0.10 was considered to show a trend toward a difference. *n* = 15 per group.

As shown in Figure 3, ADG in the OP and OPP groups was significantly lower than that in the IP and PP groups (*p* < 0.001). F:G in the OP group was significantly higher than that in the IP and PP group (*p* = 0.040).

### 3.3. Stress Hormone

**Figure 4:**
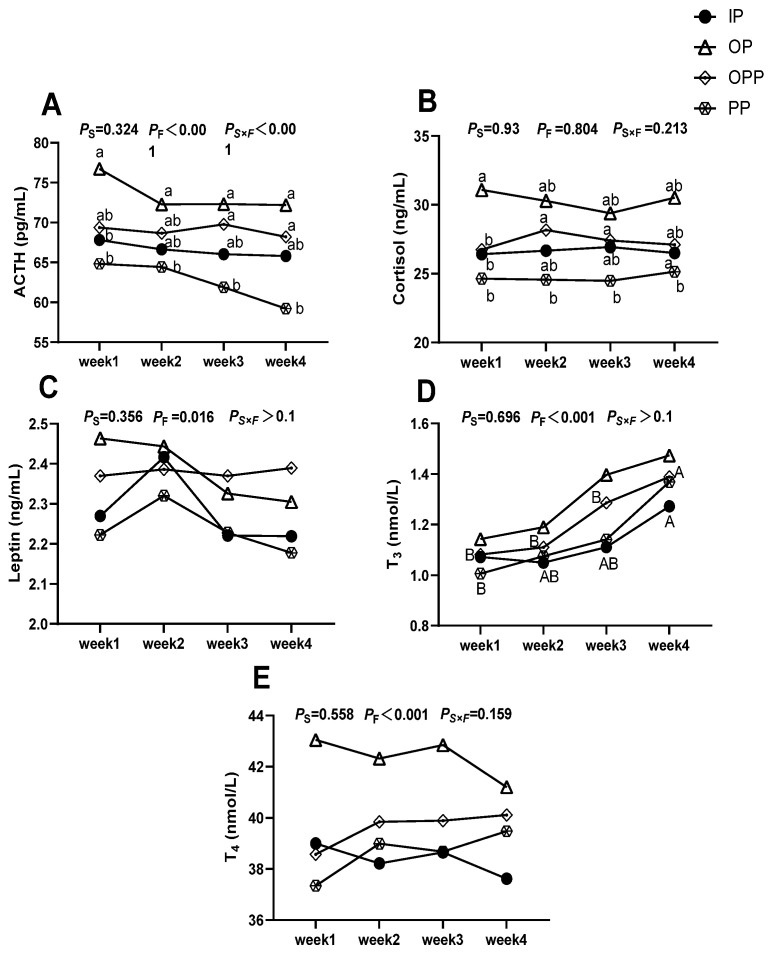
Effects of sheltering condition on serum stress hormone levels of female lambs. Note: (**A**) serumal ACTH level differences among sheltering conditions; (**B**) serumal cortisol level differences among sheltering conditions; (**C**) serumal leptin level differences among sheltering conditions; (**D**) serumal T_3_ level differences among sheltering conditions; (**E**) serumal T_4_ level differences among sheltering conditions. ACTH: adrenocorticotropic hormone; T_3_: triiodothyronine; T_4_: thyroxine. Different symbols in the lines in the figure represent different sheltering conditions. a, b: Lowercase letters (a, b) in the vertical direction indicate significant differences between sheltering conditions within the same feeding duration (*p* < 0.05); A, B: Uppercase letters (A, B) in the horizontal direction indicate significant differences between different feeding durations within the same sheltering conditions (*p* < 0.05). *P*_S_: *p*-value when sheltering conditions is the main effect, *P*_F_: *p*-value when feeding duration is the main effect. *P*_S×F_: *p*-value for the interaction between sheltering conditions and feeding duration. *p* < 0.05 indicates a significant difference, *p* < 0.01 indicates a highly significant difference, and 0.05 ≤ *p* < 0.10 was considered to show a trend toward a difference. *n* = 15 per group.

Figure 4 shows that feeding duration had a significant main effect on ACTH, T_3_, T_4_ (*p* < 0.01), and leptin (*p* < 0.05) in serum. The interaction of sheltering condition and feeding duration also significantly affected serumal ACTH. Duncan’s multiple-comparison tests further clarified the effects. Within the same sheltering condition across feeding duration, serumal T_3_ in the IP and PP groups in week 4 was significantly higher than at other time points. Comparing sheltering conditions at the same feeding duration, the OP group had significantly higher ACTH than the IP and PP groups in week 1, higher ACTH than the PP group in week 2, and both the OP and OPP groups showed significantly elevated ACTH relative to the other two groups during weeks 3–4. For cortisol, the OP group had significantly higher concentrations than all other groups in week 1, whereas the PP group had significantly lower cortisol than the other groups during week 2–4. No other comparisons reached significance.

### 3.4. Blood Biochemical Indexes

**Figure 5:**
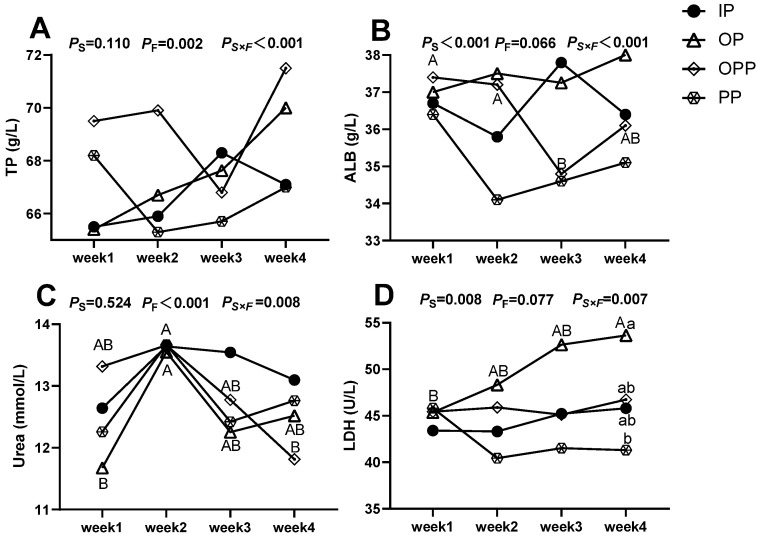
Effects of sheltering condition on serum biochemical indexes level of female lamb. Note: (**A**) serumal TP level differences among sheltering conditions; (**B**) serumal ALB level differences among sheltering conditions; (**C**) serumal urea level differences among sheltering conditions; (**D**) serumal LDH level differences among sheltering conditions. TP: total protein, ALB: albumin, LDH: lactate dehydrogenase. Different symbols in the lines in the figure represent different sheltering conditions. a, b: Lowercase letters (a, b) in the vertical direction indicate significant differences between sheltering conditions within the same feeding duration (*p* < 0.05); A, B: Uppercase letters (A, B) in the horizontal direction indicate significant differences between different feeding durations within the same sheltering conditions (*p* < 0.05). *P*_S_: *p*-value when sheltering conditions is the main effect, *P*_F_: *p*-value when feeding duration is the main effect. *P*_S×F_: *p*-value for the interaction between sheltering conditions and feeding duration. *p* < 0.05 indicates a significant difference, *p* < 0.01 indicates a highly significant difference, and 0.05 ≤ *p* < 0.10 was considered to show a trend toward a difference. *n* = 15 per group.

The effects of sheltering condition on the serum biochemical indexes level of female lambs are shown in Figure 5. When considering sheltering condition as the main effect, different sheltering conditions had a significant impact on the levels of ALB and LDH in the serum of lambs. When analyzing feeding duration as the main effect, different sheltering conditions significantly influenced TP and urea levels in serum. Additionally, the interaction between sheltering condition and feeding duration exerted a highly significant effect on the concentrations of TP, ALB, urea, and LDH in serum. Duncan’s multiple comparison test results exhibited that within the same sheltering condition across different feeding durations, the OPP group exhibited significantly higher serumal ALB levels in week 1 and week 2 than those in week 3, while serumal urea levels in week 2 were significantly lower than those in week 4. In the OP group, urea levels in week 2 were significantly higher than those in week 1, and LDH levels in week 4 were significantly elevated compared to week 1. When comparing different sheltering conditions within the same feeding duration, in week 4 the serumal LDH level was significantly higher than that of the PP group.

**Figure 6:**
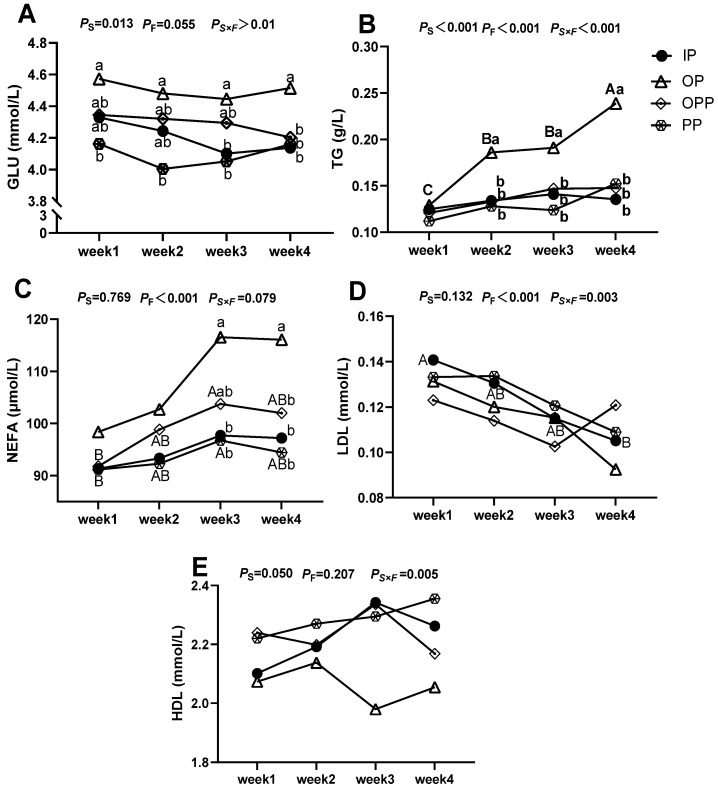
Effects of sheltering condition on serum lipid metabolism-related biochemical indexes level of female lamb. Note: (**A**) serumal GLU level differences among sheltering conditions; (**B**) serumal TG level differences among sheltering conditions; (**C**) serumal NEFA level differences among sheltering conditions; (**D**) serumal LDL level differences among sheltering conditions; (**E**) serumal HDL level differences among sheltering conditions. GLU: glucose. TG: triglyceride. NEFA: non-esterified fatty acid. LDL: low-density lipoprotein. HDL: high-density lipoprotein. Different symbols in the lines in the figure represent different sheltering conditions. a, b: Lowercase letters (a, b) in the vertical direction indicate significant differences between sheltering conditions within the same feeding duration (*p* < 0.05); A, B: Uppercase letters (A, B, C) in the horizontal direction indicate significant differences between different feeding durations within the same sheltering conditions (*p* < 0.05). *P*_S_: *p*-value when sheltering conditions is the main effect, *P*_F_: *p*-value when feeding duration is the main effect. *P*_S×F_: *p*-value for the interaction between sheltering conditions and feeding duration. *p* < 0.05 indicates a significant difference, *p* < 0.01 indicates a highly significant difference, and 0.05 ≤ *p* < 0.10 was considered to show a trend toward a difference. *n* = 15 per group.

The effects of sheltering condition on serum lipid metabolism-related biochemical indexes level of female lambs are shown in Figure 6. When sheltering condition was considered as the main effect, different sheltering conditions significantly influenced the serumal levels of GLU, TG and HDL (*p* < 0.05). When feeding duration was analyzed as the main effect, different feeding durations exerted a highly significant impact on the serumal TG, NEFA, and LDL levels in lambs (*p* < 0.01). Additionally, the interaction between sheltering condition and feeding duration exhibited a highly significant effect on the serumal concentrations of TG, LDL, and HDL (*p* < 0.01). Duncan’s multiple comparison test results exhibited that in comparisons within the same sheltering condition across different feeding durations, the OP group exhibited significantly higher serumal GLU levels than the PP group during weeks 1–2, and was significantly higher than that of the IP group and the PP group in week 3. In week 4, the GLU level in serum was significantly higher than that of all other groups. The IP group exhibited significantly higher LDL levels in serum in week 1 than in week 4, while the TG level in the serum of lamb in the OP group in week 4 was significantly higher than that during weeks 2–3, and the TG level in the serum of lamb in week 1 was significantly lower than that in other weeks. The OPP and PP groups both exhibited significantly higher serumal NEFA levels in week 3 than in week 1. For comparisons within the same feeding duration across different sheltering conditions, the OP group exhibited significantly higher serumal TG levels than all other groups during week 2–3, along with significantly elevated serumal NEFA levels compared to the IP and PP groups in week 3. By week 4, the OP group exhibited significantly higher serumal TG and NEFA levels than the other groups.

## 4. Discussion

Cold stress limits lamb production in winter. To increase feed efficiency and animal welfare, we compared the serumal hormone levels and blood biochemical parameters in lambs housed under four distinct sheltering conditions: indoor pens with enclosed housing (IP), outdoor pens (OP), house with playground pens (OPP), and polytunnel pens (PP). The duration of the study was 28 days.

### 4.1. Stress-Related Hormones of Lamb in Different Sheltering Conditions in Winter

Acute cold exposure triggers a rapid activation of the neuro-endocrine network in lambs to preserve homeostasis. This study reveals that sheltering conditions significantly influence the intensity of cold stress in lambs. Notably, the OP group exhibited the most pronounced activation of the HPA axis due to continuous exposure to low temperatures and strong winds, resulting in higher serumal levels of ACTH and cortisol compared to the PP group. Interestingly, hormone levels did not continue to rise linearly over the fourth week of cold exposure. Instead, they showed a feedback-mediated downregulation, indicating that by week 4 the lamb had developed partial cold adaptation [26].

Changes in HPA axis activity are an effective indicator of cold stress severity [27]. The paraventricular nucleus (PVN) of the hypothalamus produces stress-related hormones that stimulate the pituitary to release ACTH [27]. ACTH stimulates the adrenal cortex to increase cortisol secretion [28]. As the primary stress hormone, cortisol promotes hepatic gluconeogenesis, stimulates lipolysis, and enhances TG breakdown in adipose tissue to release fatty acids that serve as thermogenic substrates [29]. With prolonged stress exposure, however, physiological adaptation reduces HPA axis activity [30]. In our study, the ACTH and cortisol followed the same pattern. The OP group consistently showed higher weekly ACTH and cortisol levels than the PP group, which we attribute to the OP environment experiencing the lowest temperatures and highest wind speeds, resulting in a heightened stress state for the lamb. This is consistent with the research results of Xie et al. [31]. Conversely, the PP group had lower ACTH and cortisol, indicating it experienced the least cold stress.

Meanwhile, low ambient temperatures activate the HPT axis, a primary neuroendocrine pathway for thermoregulation [32,33]. In response to cold stress, the PVN secretes thyrotropin-releasing hormone (TRH), which stimulates the anterior pituitary to release TSH [34,35]. TSH then induces the thyroid gland to synthesize thyroid hormones, mainly T_4_ and the more biologically active T_3_ [36]. In our study, the observed rise in T_3_ likely reflects its role in increasing intracellular, oxidative heat production by upregulating Na^+^/K^+^-ATPase activity in cell membranes. This mechanism helps stabilize core temperature during cold exposure and mitigates the deleterious effects of low temperatures on lambs [37]. These findings agree with those of Castillo et al. [34] and Yuan et al. [38].

In addition, leptin has also been proven to be one regulator in the HPT axis function [39,40]. Leptin is a peptide hormone mainly synthesized by white adipose tissue and is positively correlated with body fat mass [41,42,43]. Leptin enhances the activity of thermogenic sympathetic nerves and accelerates the breakdown of triglycerides into energy [44]. In our experiment, leptin declined significantly in week 3 in this experiment. On the one hand, the cold environment led to an increase in fat mobilization, consuming white fat to generate energy. On the other hand, when the T_3_ level in the body rises, the body may adjust the energy balance by reducing the leptin level to prevent energy deficiency caused by excessive energy consumption in lambs.

### 4.2. Blood Biochemical Indicators of Lamb in Different Sheltering Conditions in Winter

TP is a general term for proteins in animal serum, including ALB and Globulin (GLB). ALB, the most abundant protein in animal serum, is completely synthesized by the liver [45,46] and plays an important role in maintaining colloid osmotic pressure in vivo [47]. Urea is the end product of nitrogen metabolism, and its content change represents the level of protein decomposition for energy production. In this study, the significant decline in ALB levels observed in the OPP group in week 3 corresponds with the findings reported by Hao et al. [48], which may be attributable to enhanced efficiency of protein metabolism induced by amplified cold stress responses in lambs. In addition, the urea concentration was significantly higher in week 2 of the feeding trial than in the other groups. This may be due to the influence of stress on lambs in week 2 of the feeding trial, which resulted in abnormal nitrogen metabolism in the body and an abnormal increase in urea level in serum. With continued cold stress, lambs were shown to adapt to the cold, which coincided with an increase in serum TP, and a significant decrease in urea in week 3. These results indicate that the animals initially relied on accelerated protein catabolism for energy, then developed more efficient nitrogen recycling mechanisms. This reprogramming of protein metabolism occurred alongside adaptations in carbohydrate metabolism [49]. LDH is an important rate-limiting enzyme that converts pyruvate to lactate during glycolysis and is central to cellular energy metabolism. It is often elevated in serum under such metabolic conditions [50]. In this study, the serumal LDH levels in the OP group of lambs were significantly higher than those in the other groups, with week 4 showing notably elevated LDH concentrations compared to other feeding durations. This observation may be attributed to stronger cold stress exposure in the OP group and an increased proportion of anaerobic respiration for energy supply over time, resulting in corresponding rises in lactic acid content.

When lambs are exposed to low temperatures, their sympathetic nervous system is activated, leading to the inhibition of insulin secretion and a certain degree of insulin resistance [51]. This change weakens the inhibitory effect of insulin on hormone-sensitive lipase (HSL), which is a key enzyme in lipolysis, resulting in a lipolysis increase in adipose tissue, a rapid decomposition of the TG stored in lipid droplets, and a large amount of NEFA released into the bloodstream to serve as an important energy supplement source for ruminants [52]. In this experiment, the serum NEFA level of lambs in the OP group was significantly higher than that in other groups, while that in the PP group was the lowest. This is further evidence that the cold stress intensity of lambs in the OP group was higher than that in the PP group. In week 3, the serum NEFA levels in lambs continued to rise, indicating that lambs in the OP group suffered more severe cold stress during this period.

**Figure 7:**
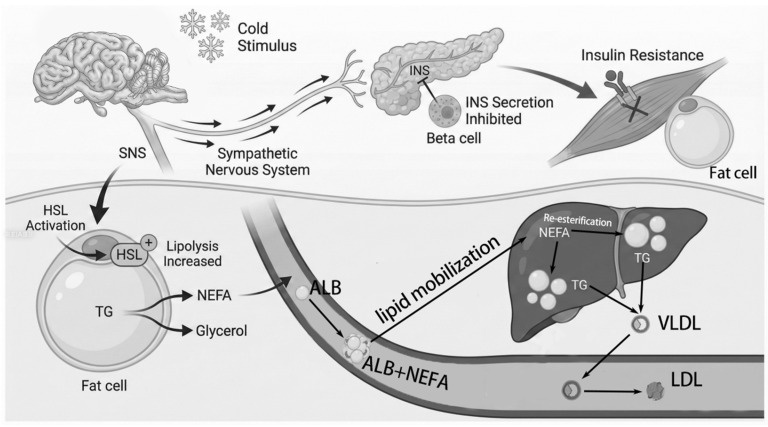
Cold stress effects on fat metabolism in lambs. Note: SNS: sympathetic nervous system, HSL: hormone-sensitive lipase, TG: triglyceride, NEFA: non-esterified fatty acid, INS: insulin, ALB: albumin, VLDL: very low-density lipoprotein, LDL: low-density lipoprotein. Cold exposure activates the SNS, which suppresses insulin secretion from pancreatic beta cells (dashed arrow with cross) and stimulates HSL (+), in adipocytes. Activated HSL hydrolyzes TG to NEFA and glycerol. NEFA bind to ALB and are transported to the liver, where they are either re-esterified to TG or used for VLDL assembly, processes that ultimately contribute to LDL formation. Thick arrows indicate neural activation; thin arrows indicate metabolic conversion or transport.

Most NEFAs released through the above-mentioned pathways bind to albumin, travel back to the liver via the circulation, and are re-esterified into TGs which are packaged into very-low-density lipoprotein (VLDL) and secreted into the bloodstream for uptake. Based on previous studies, we speculate that its mechanism conforms to Figure 7 [53,54]. VLDL is an important precursor of LDL. Generally, an increase in VLDL level elevates the LDL level and the total cholesterol level in the body. Current research suggests that when animals suffer from cold, the TG levels show a trend of first decreasing and then increasing due to the increase in energy consumption, followed by fat breakdown [55]. In this study, lambs in the OP group endured more cold stress, which increased the absorption efficiency of nutrients and the proportion of energy derived from lipolysis. LDL is a protein particle that carries cholesterol into peripheral tissues, and its main function is to transport cholesterol to tissues [56]. Generally, cold stress increases LDL levels in serum in mammals [56,57]. However, this study found that the LDL level in the serum of lambs in the IP group was significantly lower in week 4 than in week 1, which contradicts the above-mentioned pathway. Generally, cold stress causes an increase in LDL levels in the serum [58]. However, in this study, as the duration of rearing increased, the LDL levels in the lamb’s serum significantly decreased. The underlying mechanism may involve enhanced free fatty acid metabolism coupled with suppressed VLDL synthesis. Specially, the reason for this might be that during long-term cold stress, a large amount of FFA is rapidly transported to the liver for beta-oxidation of fatty acids, generating a large amount of heat. At this time, the large amount of FFA flowing into the liver inhibits the synthesis and secretion of VLDL in the liver [59,60]. And VLDL is the precursor particle of LDL, which may have caused the decrease in LDL levels in the serum of lambs under long-term cold stress [59,61]. Meanwhile, cold increases feed intake but decreases its digestibility, thus affects the supply of substrates required for liver lipoprotein synthesis, and ultimately decreases lipoprotein synthesis. And the consumption of TG increases under continuous energy demand, which limits LDL.

In addition, GLU is the primary immediate energy substrate, and its blood concentration directly reflects systemic energy status. In this experiment, serumal GLU concentration in the OP group was higher than in the other groups, consistent with Sasaki et al. [62]. It likely reflects a compensatory hyperglycemic response to cold stress. Reduced insulin secretion limits both peripheral GLU uptake and promotes hepatic glycogenolysis/gluconeogenesis, to maintain energy supply. Meanwhile, except in the first week of the feeding trial, the TG level in the OP group was always significantly higher than that in the other groups, indicating that it had entered a stable mobilization stage, reflecting higher nutrient absorption efficiency and an up-regulated proportion of fat for energy supply. This trend of first decreasing and then increasing was in line with the stage characteristics of energy metabolism under cold exposure. The aforementioned content further substantiates that the lambs in the OP group experienced a more pronounced state of cold stress, whereas those in the PP group exhibited a milder level of cold stress.

### 4.3. Limitation

To enable rapid and cost-effective barn monitoring, we measured multiple gas concentrations using detector tubes. Although these tubes offer limited precision, they consistently distinguish the different gas environment characteristics among the animal houses examined. In addition, this study was conducted on a single ranch in Hohhot, central Inner Mongolia; subsequent trials in other regions are needed to verify its generalizability.

## 5. Conclusions

At the early stage of cold stress, energy was generated through enhanced fat decomposition and protein metabolism, whereas in the later stage, the lambs started to store energy and reduce fat and protein decomposition as they acclimated to the low temperature. Comprehensive analysis of all data showed that, during the four-week feeding period, the OP group experienced the highest stress levels, while the PP group had the lowest under the experimental conditions. However, in the PP group, poor ventilation and higher temperatures led to ammonia accumulation in the pens, which may adversely affect lamb growth and health. In conclusion, under adequate ventilation, this experiment found that among the four sheltering conditions, PP was the most suitable option for winter feeding of lambs in Inner Mongolia. Further study is required to determine the balance between ventilation rates in PP sheds and the resulting indoor temperature.

## Figures and Tables

**Table 1:** Composition and nutrient levels of the experimental diet (air-dried basis).

Item	Content	Item	Content
Ingredients		Nutrient levels	
Corn/%	14.58	Digested energy/(MJ/kg) ^②^	11.12
Foxtail millet stover (air-dried)/%	50.00	DM/%	90.72
Alfalfa hay/%	8.80	CP/%	13.62
Cottonseed meal/%	8.26	EE/%	1.96
Soybean meal/%	5.30	NDF/%	44.08
Wheat bran/%	8.36	ADF/%	29.08
DDGS ^③^/%	3.30	Ash/%	8.01
CaHPO_4/_%	0.12	Ca/%	0.83
NaCl/%	0.30	P/%	0.41
NaHCO_3_/%	0.48		
Premix ^①^/%	0.50		
Total/%	100		

Note: ① The premix provided the following nutrients per kg of diets: vitamin A 6000 IU, vitamin D3 2000 IU, vitamin E 15 IU, vitamin K3 1.8 mg, vitamin B1 0.35 mg, vitamin B2 8.5 mg, vitamin B6 0.9 mg, vitamin B12 0.03 mg, D-pantothenic acid 16 mg, nicotinic acid 22 mg, folic acid 1.5 mg, biotin 0.15 mg, Cu 8 mg, Fe 40 mg, Mn 20 mg, Zn 40 mg, I 0.8 mg, Se 0.3 mg, Co 0.3 mg. ② DE was a calculated value, while the others were measured values. ③ DDGS: distillers dried grains with soluble, DM: dry matter, CP: crude protein, EE: ether extract, crude fat, NDF: neutral detergent fiber, ADF: acid detergent fiber, Ash: coarse ash, Ca: calcium, P: phosphorus.

## Data Availability

The original contributions presented in this study are included in the article. Further inquiries can be directed to the corresponding authors.
